# The potential relationship between Flammer and Sjögren syndromes: the chime of dysfunction

**DOI:** 10.1007/s13167-017-0107-5

**Published:** 2017-08-14

**Authors:** Babak Baban, Olga Golubnitschaja

**Affiliations:** 1 Department of Oral Biology, Dental College of Georgia, Augusta University, 1120, 15th St, CL 2140, GA 30912 Augusta, USA; 2 Department of Surgery/Section of Plastic Surgery, Augusta University, 1120, 15th St, CL 2140, GA 30912 Augusta, USA; 3 Department of Neurology, Medical College of Georgia, Augusta University, 1120, 15th St, CL 2140, GA 30912 Augusta, USA; 4 Radiological clinic, Rheinische Friedrich-Wilhelms-University of Bonn, Sigmund-Freud-Str 25, D-53105 Bonn, Germany; 5 Breast Cancer Research Centre, Rheinische Friedrich-Wilhelms-University of Bonn, Bonn, Germany; 6 Centre for Integrated Oncology, Cologne-Bonn, Rheinische Friedrich-Wilhelms-University of Bonn, Bonn, Germany

**Keywords:** Predictive preventive personalied medicine (PPPM), Health policy, Flammer syndrome, Sjögren syndrome, Patient stratification, Vascular dysregulation, Autoimmune disease, Gender, Symptom, Genetic, Environment

## Abstract

Flammer syndrome (FS) is a term to blanket a cluster of vascular and nonvascular signs and symptoms linked to primary vascular dysregulation (PVD), increased sensitivity to various stimuli (stress, drugs, etc.) and altered sense regulation such as pain, smell and thirst perception. On one hand, disruption of blood barrier and homeostasis of the body are the main targets of vascular irregularity. Inflammation and immune disorders including autoimmunity are considered as a consequence of the abnormal vascular regulation processes. On the other hand, decreased thirst feeling typical for FS-affected individuals may lead to extensive body dehydration resulting in dry eye appearance and breast cancer (BC) risk, amongst others. To this end, recent research demonstrated FS as linked to BC development and progression into the metastatic disease. On the other side, Sjögren syndrome (SS) is an autoimmune disease characterised by a progressive sicca syndrome associated with the dry eye symptoms, specific immunologic complex and/or significant infiltrate at minor salivary gland biopsy. SS is relatively frequent, with a clinical diagnosis predominantly amongst women. Its physiopathology is a complex battery of both environmental and genetic factors. If left untreated, SS may be associated with and/or resulted in severe arthritis and the development of B cell lymphoma. In this mini-review, we summarise the facts and hypotheses connecting FS and SS symptoms together and mechanisms potentially overlapping in both syndromes. Unraveling the common denominators between these two syndromes not only providing more evidence for interaction between altered sense regulation, vascular dysregulation, immune system dysfunction but also focusing on the individual outcomes in terms of severity grade and potential complications exploring novel diagnostic, prognostic and treatment modalities. Multi-professional considerations presented here are an example how to effectively enter the new era of preventive, predictive and personalised medicine benefiting the patients and healthcare system as the whole.

## Introduction

Flammer syndrome (FS) is defined as a combination of symptoms resulting from a predisposition to a generally increased sensitivity to stimuli. Compared to the general population, FS-affected individuals react differently to environmental stimuli, such as cold and physical or emotional stress. Nearly all organs, particularly the eye, can be involved indicating the systemic effects by FS [1–3]. FS is characterised by strongly pronounced primary vascular dysregulation (PVD) along with a cluster of symptoms and signs that may occur as the suboptimal health condition in healthy individuals as well as in several patient cohorts investigated such as neurodegenerative disorders (glaucoma and multiple sclerosis), cancer and metastatic disease [4–9].

Although the syndrome has some protective effects against the development of atherosclerosis, however, FS presents an increased frequency of optic disc hemorrhages, activated retinal astrocytes, elevated retinal venous pressure, optic nerve compartmentalisation, fluctuating diffuse visual field defects, elevated oxidative stress, and systemic hypoxia impacting individual outcomes in several pathologies such as cancer and metastatic disease [1, 7–9].

Sjögren syndrome (SS) is a systemic chronic inflammatory disorder characterised by an impaired edothelium-dependent vasodilation in primary SS patients [10] and lymphocytic infiltrates into exocrine organs. Most individuals with Sjögren syndrome present with sicca symptoms, such as xerophthalmia (dry eyes), xerostomia (dry mouth), and parotid gland enlargement. While, primary Sjögren syndrome (PSS) occurs in the absence of another underlying rheumatic disorder, secondary Sjögren (SSS) syndrome is associated with additional underlying rheumatic disease, such as systemic lupus erythematosus (SLE), rheumatoid arthritis (RA), or scleroderma. Given the overlap of Sjögren syndrome with many other disorders, it is plausible to explore the potential reciprocal relationship between SS and FS [11–13].

In addition to a number of epidemiologic common denominators, both FS and SS show cardiovascular dysfunction at different levels as they progress during the course of diseases [1, 12, 14]. In fact, while a direct correlation between FS and retinal venous pressure (RVP) in patients with glaucoma has already been shown, it is also reported that SS patients may be more vulnerable to glaucoma. Similarly, several studies have indicated a relationship between optic neuritis and initial presentations of both FS and SS. Further, some researchers have demonstrated cardiac arrhythmias and vascular dysfunction as the initial manifestations of adult primary Sjögren’s syndrome [14, 15].

**Fig. 1:**
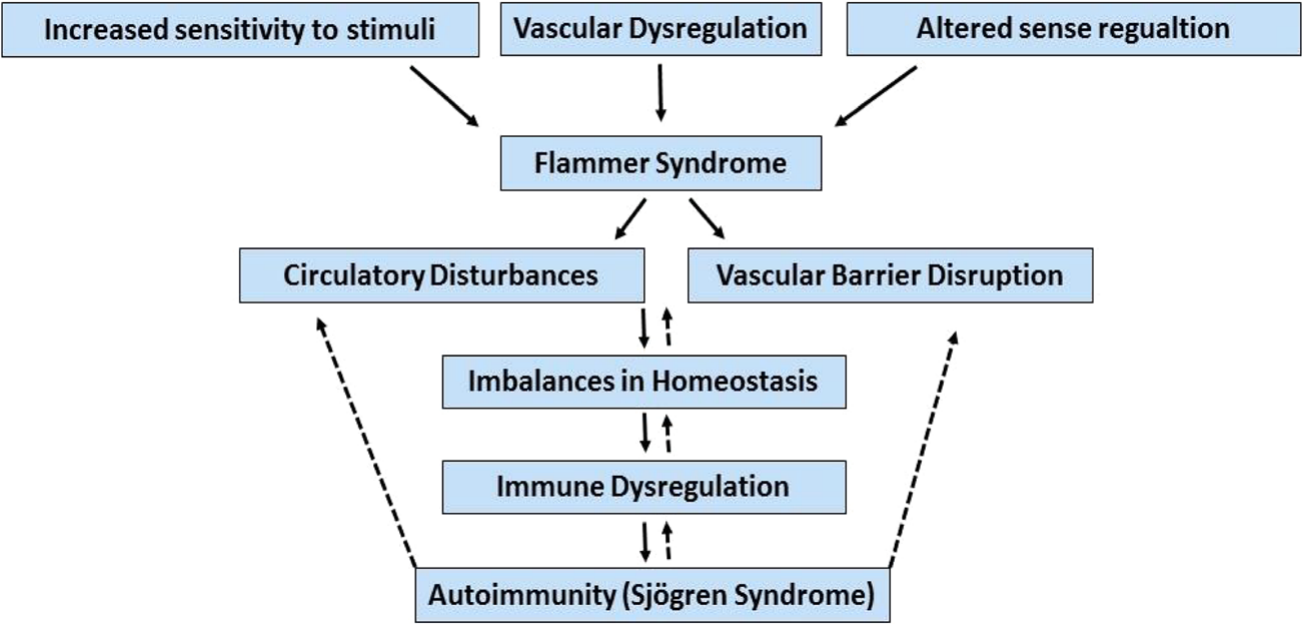
The schematic potential relationship between Flammer syndrome and Sjögren syndrome

In this present mini-review, we highlight the findings that reveal some resemblances between FS and SS. These common mechanisms not only help clinicians with potential novel therapies for treating both FS and SS, but also may provide appropriate tools in the context of predictive, preventive and personalised medicine as the medicine of the future (Fig. 1).

## Flammer syndrome

Flammer syndrome (FS) is a relatively recently described health condition [1] linked to primary vascular dysregulation (PVD), increased sensitivity to various stimuli (stress, drugs, etc.) and altered sense regulation such as pain, smell and thirst perception. It is represented by a cluster of symptoms and signs that can occur as a sub-optimal health condition in healthy individuals as well as patient cohorts. The leading symptoms include cold hands and/or feet, low blood pressure, prolonged sleep onset time, shifted circadian rhythm, increased sensitivity to pain, enhanced smell perception, and reduced feeling of thirst. Although FS individuals are typically less thirsty, only those who are aware of being affected by FS do drink enough controlling their water intake by head. However, FS-affected individuals who are not aware of these deficits are strongly predisposed to the whole body dehydration that may result in several related pathologies such as dry eye and breast cancer [16]. With higher prevalence in females, FS incidence is associated with certain physical, occupational and psychological conditions. As such, FS is seen more in slender than in obese individuals and in subjects with systemic hypotension more than in subjects with hypertension. Demographically, FS is more prevalent in people with indoor jobs than in those with outdoor jobs, in academics more than in blue collar workers, and in Asians more often than in Caucasians [1]. The symptoms appear early in life with puberty and mitigate with progressing age, in women specifically after menopause. FS individuals frequently indicate that one or both parents suffered from the same syndrome. Therefore, a genetic component is likely to be involved in the molecular mechanisms which underlie FS [1, 15].

Vascular dysregulation is the basic feature of FS. Importantly, it is reported that FS is associated with or predisposes to the development of diseases such as normal tension glaucoma and often contribute to low blood pressure [1, 17]. These notions were supported by the clinical findings that glaucoma patients with FS often had dilated retinal veins, suggesting the retinal venous pressure (RVP) in the subset of glaucoma patients with FS may be higher than in those glaucoma patients without FS. As a mechanism responsible for such higher RVP, it has been propounded that in FS patients retinal astrocytes are more often activated, oxidative stress is increased and optic nerve compartment syndrome could more often be detected [1]. Moreover, in the patients with FS, not only the retinal vessels of the optic nerve head are less shifted to the nasal side, but also larger long-term fluctuations of the diffuse component of visual field defects are observed [1–3]. All these suggest that the vascular systems of people with FS respond differently to various stimuli (e.g. reacting with vasoconstrictions to cold or stress) [1, 18, 19]. Despite the anatomically normal appearance of their vessels, those people with FS have stiffer retinal vessels, as pulse waves in their retinal vessels propagate faster compared to those of subjects without FS [1, 20].

## Sjögren syndrome

Sjögren syndrome (SS) is a systemic autoimmune disease with a prevalence of 1–3%, characterised by an impaired endothelium-dependent vasodilation in primary SS patients [10], affecting more women than men (ratio of 9:1). SS is characterised by lymphocytic inflammation of lachrymal and salivary glands resulting in dryness of the mouth and ocular mucosa as well as polyclonal B lymphocyte hyperactivity with a characteristic autoantibody profile (rheumatoid factors, anti-SS-A and anti-SS-B antibodies). Clinically, in addition to the cardinal presentation of sicca symptoms of dry eyes followed by dry mouth, other common presenting manifestations of Sjögren syndrome may, therefore, include inflammatory joint and muscle pain, chronic fatigue, swollen salivary glands, demyelinating disease, neuropathies and abnormal lab values. In the pediatric population, the most common presentation of childhood SS is recurrent parotitis [12, 21, 22]. Onset of the SS usually occurs in young women, and a benign course of the disease is often encountered [23]. The salivary gland dysfunction is of major consequence for oral health including increased susceptibility to dental caries, gingivitis, and periodontitis [12, 24–26]. SS can occur as a clinical entity alone or co-expressed with other systemic autoimmune rheumatic disorders. The serological hallmark of SS is the presence of circulating autoantibodies against soluble nuclear RNA containing antigens, Ro/SSA, and La/SSB [12, 27, 28].

The aetiology and pathogenesis of SS remain elusive. Although activation of innate immunity and infiltration of lymphocytes (B and T cells) are considered as the histo-pathologic hallmark of SS, however, increasing evidence suggest that immune dysfunction is not the sole mechanism underneath local and systemic complications of SS. A collection of cardiovascular irregularities including renal (e.g., glomerulonephritis), cardiac and neurologic dysfunctions may occur both prior and/or post to the onset of SS [29–33]. Thus, it is essential to unravel the contribution of the endogenous mechanisms which regulate local tissue inflammatory environment and could also contribute to the recruitment of immune and inflammatory cells with consequent further exacerbation of the disease process.

## Common denominators between FS and SS: new paradigm of synergic dysfunction and potential modality in treating immune-vascular disorders

The relationship between symptoms of FS and certain vascular dysregulation-derived and systemic hypoxic effects impacted diseases such as (but not limited to) multiple sclerosis (MS) [6] glaucoma, and breast cancer with aggressive metastatic disease have been already reported [1, 2, 7–9, 17]. Although a number of studies have proposed a protective role for FS against the development of atherosclerosis (ATS), however, the contribution of FS to the eye diseases such as glaucoma and retinitis pigmentosa has been already shown [1, 34]. Importantly, glaucoma patients with FS have additional signs and demonstrate an unilateral nonrecurring choroidal infarction, and a chronic progressive bilateral glaucomatous optic neuropathy [1, 35]. In fact, FS is considered to be a risk factor for both occlusions of ocular vessels and glaucomatous optic neuropathy [28]. Generally, patients who develop glaucomatous damage despite a normal IOP or patients with progressing glaucomatous damage despite well-controlled IOP very often suffer from Flammer syndrome. Glaucoma patients with FS have particularly large long-term fluctuations of the diffuse component of visual field defects, which is best observed with the help of a Bebie curve [1, 22]. Noteworthy, FS-affected individuals demonstrated shifted expression patterns in circulated leukocytes that indicate an involvement of the immune system in pathogenesis of FS [4, 5].

It has been reported that patients with SS, especially those with increased positivity of autoantibodies, might be prone to developing glaucoma when exposed to other glaucomatous risk factors, such as increased IOP or vascular dysregulation. Peripapillary retinal nerve fiber layer (pRNFL) thickness, macular ganglion cell-inner plexiform layer (mGCIPL) thickness, and optic nerve head parameters were compared between control groups and patients with SS. It was revealed that eyes of SS showed thinning of pRNFL and mGCIPL thicknesses compared to the control group [15, 36–39]. Although SS patients were not clinically regarded as having glaucomatous optic neuropathy, however, the degree of thinning correlated with increased numbers of the positive autoantibody suggesting that SS patients might be prone to develop glaucoma when exposed to other glaucomatous risk factors such as increased IOP or vascular dysregulation. These findings should be considered when diagnosing or evaluating glaucomatous structural changes in SS patients [1, 15, 38–40]. Importantly, it is known that SS often coexists with other systemic autoimmune diseases, including (not limited to) RA and SLE [40–42]. In fact, it can be at any stage of SS when patients have another well-defined major connective tissue disease, in particular, RA and systemic lupus erythematosus [40]. Interestingly, dry eye disease (DED), as one of the main complications associated with SS, is also common in RA patients [39].

To summarise, although it is still too premature to make a definite relationship between FS and SS, but it is plausible to suggest such a connection, due to a number of symptoms and signs in common. Despite the fact that the mechanisms responsible for such concordance are not yet understood, however, it is reasonable to propose that a systemic vascular dysregulation and consequent functional impairment of the wall of peripheral arteries and vasculatures may facilitate the initiation, development and progression of autoimmune diseases including SS. A combination of chronic inflammation and immunological factors may explain the dysfunction of endothelium and vascular smooth muscle cells during the course of FS and SS, supporting the potential concerted symptoms and consequences, plugging FS (e.g. genetically predisposed individuals with particularly pronounced FS phenotype) into SS. Table 1 contains some of the common clinical manifestations of FS and SS.

**Table 1:** Common clinical symptoms between Flammer syndrome and Sjögren syndrome

Clinical features	Flammer syndrome	Sjögren syndrome
Gender prevalence (higher in female)	Yes	Yes
Changes in blood supply	Yes	Partially
Changes in blood barrier permeability	Yes	Yes
Association with other systemic autoimmune diseases	Yes	Yes
Modulation of immune system	Yes	Yes

## Concluding remarks: a preventive, predictive and personalized perspective

Vascular dysfunction is a multifactorial phenomenon known as the basis for many disorders and their consequential complications. The term Flammer syndrome (FS) was introduced to blanket a suboptimal health condition with a characteristic cluster of vascular and nonvascular signs and symptoms. FS is involved in the pathology of or even may predispose to a spectrum of diseases such as normal tension glaucoma, retinal vein occlusion in patients without classical risk factors, sudden hearing loss, dry eye, breast cancer and metastatic disease, amongst others [7–9, 15].

Sjögren syndrome (SS), a systemic autoimmune disease, shares a number of signs, causative factors and abnormalities with FS. Hence, FS-induced vascular dysregulation disturbs the homeostasis of circulation which in turn may result in inflammatory responses and immunologic disorders, leading to the autoimmune diseases such as SS. Further, typical for FS decreased thirst feeling, if remaining uncontrolled, may result in the whole body dehydration with all potential consequences such as dry eyes, nose, mouth, cavities, skin as well as vaginal dryness and liver problems, amongst others - the symptoms charactetistic for SS that should be reciprocally investigated in FS and SS. Therefore, better understanding of mechanisms responsible for the pathophysiology of FS and SS, targeting common features and defining interaction between them would establish more reliable platform to launch individualised preventive and predictive measures effectively [43] against further progression of both FS and SS and development of potential follow-up pathologies. Hence, further studies are needed not only to identify functional links between FS and SS but also to confirm initial findings, clarify the meaning of these associations and translate them into PPPM-guidelines [43] to foster updated health policy, higher standards of health care and life quality of affected patient cohorts.
